# Soiled airway tracheal intubation and the effectiveness of decontamination by paramedics (SATIATED): a randomised controlled manikin study

**DOI:** 10.29045/14784726.2019.06.4.1.14

**Published:** 2019-06-01

**Authors:** Richard Pilbery, M. Dawn Teare

**Affiliations:** Yorkshire Ambulance Service NHS Trust; University of Sheffield

**Keywords:** airway obstruction, emergency medical services, intubation

## Abstract

**Introduction:**

Vomiting and regurgitation are commonly encountered in out-of-hospital cardiac arrest (OHCA), with a reported incidence of 20–30%. This is of concern since patients who have suffered an OHCA are already in extremis. If standard suctioning techniques are not sufficient to maintain a clear airway and provide ventilation, then these patients will die, irrespective of the quality of chest compressions and the timeliness of defibrillation. This study aimed to determine whether a short teaching session of the suction assisted laryngoscopy and airway decontamination (SALAD) technique improved paramedics’ ability to successfully intubate a contaminated airway.

**Methods:**

A modified airway manikin with the oesophagus connected to a reservoir of ‘vomit’, and a bilge pump capable of propelling the vomit up into the oropharynx, was used to simulate a soiled airway. The intervention consisted of a brief SALAD training session with a demonstration and opportunity to practice. Participants were randomly allocated into two groups: AAB, who made two pre-training intubation attempts and one post-training attempt, and ABB, who made one pre-training and two post-training attempts, to adjust for improvement in performance due to repetition.

**Results:**

In this manikin study, following a brief SALAD training session, more paramedics were able to intubate a soiled airway on their first attempt, compared to those without training (90.2% vs. 53.7%, difference of 36.6%, 95% CI 24–49.1%, p < 0.001). In addition, the mean difference in time taken to perform a successful intubation between groups was statistically significant for attempts 1 and 2 (mean difference 11.71 seconds, 95% CI 1.95–21.47 seconds, p = 0.02), but not attempts 1 and 3 (mean difference –2.52 seconds, 95% CI –11.64–6.61 seconds, p = 0.58). This result is likely to be confounded by the use of tracheal suction, which only occurred in the post-training attempts and added additional time to the intubation attempts. There was no statistically significant difference in success rates on the third attempt between AAB and ABB (89.0% vs. 86.6%, difference 2.4%, 95% CI 7.6–12.4%, p = 0.63).

**Conclusion:**

In this study, the use of the SALAD technique significantly improved first attempt success rates when paramedics were intubating a simulated soiled airway.

## Introduction

Vomiting and regurgitation are commonly encountered in out-of-hospital cardiac arrest (OHCA), with a reported incidence of 20–30% (Benger et al., 2018; Simons, Rea, Becker, & Eisenberg, 2007; Voss et al., 2014). This is of concern since patients who have suffered an OHCA are already in extremis and there is limited evidence that the presence of emesis leads to a reduction in odds of survival (Simons et al., 2007). If standard suctioning techniques are not sufficient to maintain a clear airway and provide ventilation, then these patients will die, irrespective of the quality of chest compressions and the timeliness of defibrillation. Arguably, tracheal intubation is the preferred airway management technique in patients with ongoing airway contamination, but there is evidence that this is difficult to achieve when the airway is soiled (Sakles et al., 2017). Early intubation may also help prevent aspiration pneumonias which are thought to adversely affect survival outcome, although this has yet to be proved empirically (Christ et al., 2016).

Traditional suctioning techniques have been criticised, and paramedic training in the management of contaminated airways, limited. This has led to the development of a combined suction/laryngoscopy technique to facilitate intubation, known as suction assisted laryngoscopy and airway decontamination (SALAD), and the creation of modified airway manikins to allow for practice in these techniques (DuCanto, Serrano, & Thompson, 2017).

However, to date there has only been one study specifically looking at the SALAD technique, and the outcomes were limited to self-reported confidence measures of trainees using the technique. Other techniques have been described to manage significant airway contamination, including the use of a meconium aspirator (Kei & Mebust, 2017), which requires a device that is not typically carried by UK ambulance services, and deliberate intubation of the oesophagus (the oesophageal diversion manoeuvre), of which the sum total of evidence in support of the procedure is a single case report (Kornhall, Almqvist, Dolven, & Ytrebø, 2015).

This study aimed to determine whether a short teaching session of the SALAD technique to paramedics improved their ability to successfully intubate a contaminated airway. The primary objective was to determine the difference between paramedic first-pass intubation success before and after SALAD training, in a simulated soiled airway. The secondary objective was to determine the difference in time taken to achieve first-pass intubation success, before and after SALAD training in a simulated soiled airway, since it was hypothesised that the time to successful intubation might improve as a result of repeated attempts at intubation – that is, be due to a practice effect, rather than the SALAD technique alone.

## Methods

### Study design and participants

This randomised controlled trial was conducted in the Yorkshire Ambulance Service NHS Trust (YAS) between August and December 2018. Participants were NHS staff employed by YAS, who were Health and Care Professions Council (HCPC) registered paramedics at the time of enrolment in the study, were authorised to intubate and who had received no SALAD training in the previous three months. Potential participants were excluded if they did not meet the inclusion criteria, were allergic to the ‘vomit’ ingredients or were unwilling to provide consent to participate.

### Randomisation

In order to adjust for improvements in participant performance that might arise from repeated attempts at intubation, beyond that of the SALAD training itself, paramedics were randomised into either: making two pre-training intubation attempts and one post-training attempt (group AAB); or making one pre-training intubation attempt and two post-training attempts (ABB). Groups were evenly allocated (i.e. 1:1) using a block randomisation sequence provided by RANDOM.ORG. To distinguish between the training pathways and number of the assessed attempts, group AAB’s attempts were denoted A_01_A_02_B_01_ and group ABB’s, A_11_B_11_B_12_. It was not possible to blind participants or the researcher from their individual allocation.

### Intervention

#### SALAD manikin

**Figure 1:**
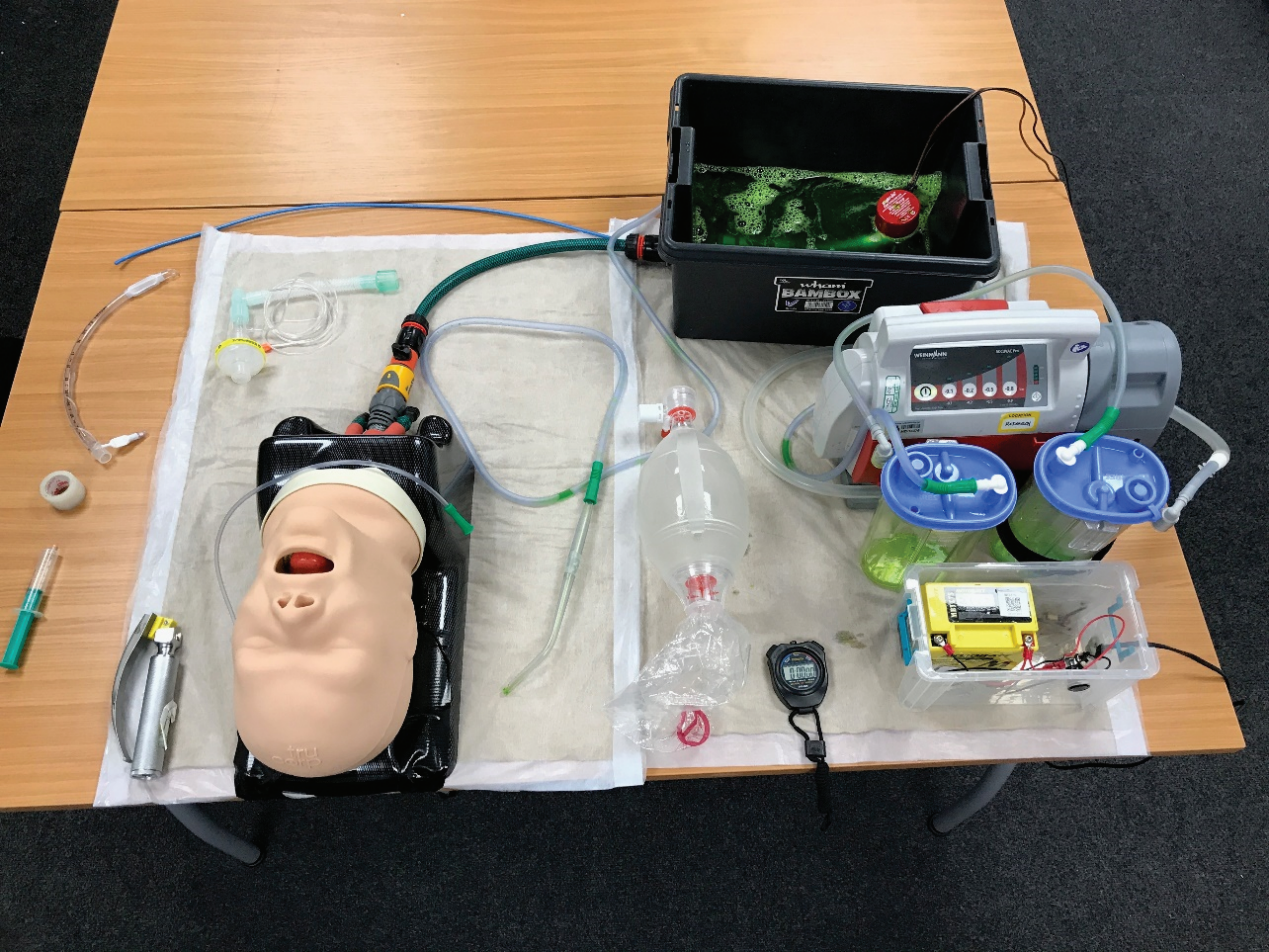
Figure 1. SALAD manikin set-up used for the study.

A modified TruCorp AirSim Advance airway manikin was used for the study as it has realistic airway anatomy and can be used for tracheal intubation training (Yang et al., 2010). The oesophagus of this manikin was connected, via a hosepipe, to a bilge pump sited within a reservoir of simulated vomit (Figure 1). The vomit was water, coloured with food-grade colouring and thickened with xanthan gum (a food additive). While there are recipes for solid-containing vomit, it was decided that as a first introduction to the technique, thickened opaque vomit would present a sufficient challenge. Once the bilge pump was switched on, a constant flow of vomit was propelled into the oropharynx, obscuring any view of the laryngeal inlet. The flow rate was controlled by a tap, which was calibrated to provide 1 L/min of vomit to the oropharynx of the manikin during intubation attempts. To keep vomit within the oropharynx, the left and right bronchi on the manikin were occluded.

Standard intubation equipment, including personal protective equipment (PPE) and motorised suction routinely used within YAS, was provided for participants, and the study researcher acted as a competent assistant for the intubation attempts.

#### Procedure

Once informed consent was obtained, paramedics were randomised into either group AAB or ABB. All attempts utilised direct laryngoscopy, which is the standard intubation technique within YAS. Prior to each intubation attempt, the manikin was primed with vomit to ensure the same level of oropharyngeal obstruction. All attempts were video recorded for timing accuracy.

Participants were deemed to have commenced their attempt once the bilge pump was turned on. The attempt was considered to be over when either: the paramedic intubated the manikin and verbally confirmed with the researcher that the attempt had been completed; or, 90 seconds had elapsed; or, the tracheal tube was placed into the oesophagus and the cuff inflated while the pump was still running.

If the tracheal tube was not in the trachea, with the cuff inflated and connected to a bag-valve device within 90 seconds, the attempt was considered a failure. While it is generally advocated that intubation attempts should take no longer than 30 seconds, this assumes that it is possible to pre-oxygenate patients before an intubation attempt, and re-oxygenate them in the event that intubation is not possible. In patients who have an oropharynx full of vomit, oxygenation is not possible. Therefore, a pragmatic and prolonged target of 90 seconds was suggested by an internationally recognised SALAD expert, Dr James DuCanto (J. DuCanto, personal communication, 26 April 2018).

Participants randomised into the two pre-training attempts group (AAB) made their second intubation attempt immediately following the first, and prior to the group training session. Once all participants completed their pre-training intubation attempt(s), the training session was delivered. The training intervention adopted the Advanced Life Support Group/Resuscitation Council 4-stage approach of skills teaching (Bullock, Davis, Lockey, & Mackway-Jones, 2008), comprising:

1. a real-time demonstration of the SALAD technique by the researcher;
2. a repeated demonstration with an explanation of the rationale of the steps taken when performing SALAD (not real-time);
3. another demonstration of the SALAD technique conducted by the researcher, but guided by one of the participants; and
4. an attempt by the same participant who guided the researcher in the previous step, followed by a practice attempt by the other participants.

Following the training session, participants made their post-training intubation attempt(s) using the same method as for the pre-training intubation attempt(s). Participants randomised into the two post-training attempts (ABB), made their second attempt immediately following the first post-training attempt.

### Outcomes

The primary outcome was the difference in proportions of paramedic first-pass intubation success rates, before and after SALAD training.

The secondary outcomes were:

- mean of the differences between groups AAB and ABB with respect to the first and second successful intubation attempt times, and between the first and third successful intubation attempt times, in order to detect improvements in time to successful intubation; and
- difference in success rates between participants who had two post-training intubation attempts (ABB) versus participants who only had one post-training intubation attempt (AAB).

### Statistical analysis

#### Sample size

A sample size of 154 participants was calculated to be required to detect a change in the proportion of intubation successes, from 0.25 in the pre-training group, to 0.50 in the post-training group, with a power (1-β) of 90% and a significance level (α) of 5%. Given that there is no literature to guide expected performance, a conservative estimate was made in consultation with Dr James DuCanto (J. DuCanto, personal communication, 26 April 2018).

#### Primary outcome analysis

To determine if the training had an effect and increased the success rate of intubation, the proportions of success in the groups that received no training before their second intubation attempt (A_02_) was compared to those who did receive training before their second intubation attempt (B_11_). Comparing the rates at these time points controlled for any learning effect due to participants making more than one attempt at intubation. The difference in the two proportions was analysed using a two independent samples proportion z-test, assuming a two-sided type 1 error rate of 5%.

#### Secondary outcome analysis

Only successful intubation attempts and their timings were included in the secondary outcome analysis. It was hypothesised that time to successful intubation might improve as a result of repeated attempts at intubation – that is, be due to a practice effect, rather than the SALAD technique alone. Therefore, the mean of the attempt time differences A_01_–A_02_ was compared with the mean of attempt time differences A_11_–B_11_. In addition, the mean of the attempt time differences seen at the final measurements (A_01_–B_01_), was compared to the mean of the attempt time differences A_11_–B_12_. Finally, success rates between B_01_ and B_12_ attempts were compared to see whether practice following training improved the intubation success rate. A student’s t-test was utilised to test for the differences between mean pre- and post-training intubation attempt times, and a two independent samples proportion z-test to test the difference in success rates.

## Results

A total of 164 participants took part in SATIATED, with an equal number in groups AAB and ABB. The groups were similar with respect to intubation attempts (successful or not) undertaken in the previous 12 months. The median number of years as a paramedic was 1.5 years less in group ABB, although the interquartile range was similar. Of the participants, 36 had heard of the SALAD technique prior to the study, with a slightly higher number in group ABB (Table 1).

**Table 1.** Summary details of participants.

Measure	AAB	ABB	Total
**n**	82	82	164
**Median intubation attempts in past 12 months (IQR)**	2.5 (0–6)	3.0 (1–7)	3.0 (1–6.5)
**Median number of successful intubation attempts in past 12 months (IQR)**	2 (0–5)	2 (0–6)	2 (0–6)
**Median years as paramedic (IQR)**	5.0 (1–10)	3.5 (0–10)	4.0 (1–10)
**Aware of the SALAD technique**	15	21	36

**Figure 2:**
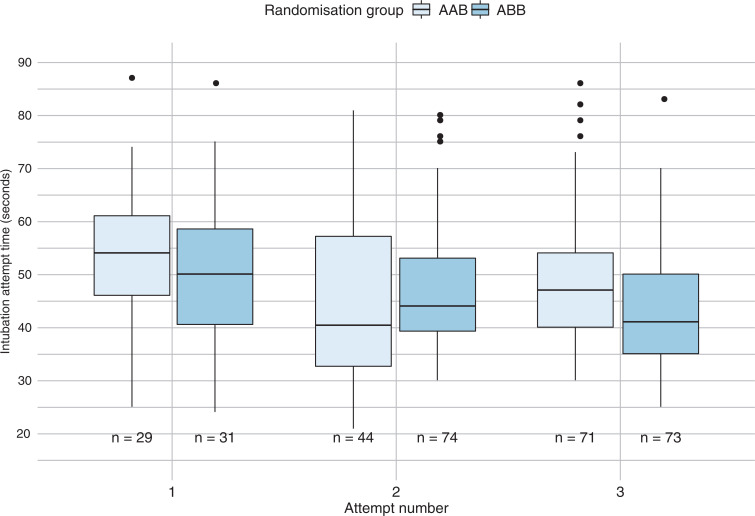
Figure 2. Successful intubation attempt times, stratified by randomisation sequence and attempt number.

Note: N = 82 for each group (AAB/ABB) and attempt.

First-pass intubation success with and without SALAD on the second attempt was 90.2% and 53.7%, respectively (Table 2) – a significant difference of 36.6% (95% CI 24–49.1%, p < 0.0001). Figure 2 summarises the successful intubation attempt times by participants in each randomisation group. For successful intubation attempts, group ABB was generally faster except on attempt 2, where AAB intubated sooner (Table 2).

**Table 2.** Summary data of the differences between successful intubation attempts.

	Attempt 1		Attempt 2		Attempt 3	
Measure	AAB	ABB	AAB	ABB	AAB	ABB
Successful attempts n (%)	29 (35.4)	31 (37.8)	44 (53.7)	74 (90.2)	71 (86.6)	73 (89)
Median elapsed time to intubation attempt (secs) (IQR)	7 (4–13)	6 (3–11)	4 (2–8.5)	4 (2–6)	4 (2–5.5)	3 (2–5)
Median intubation attempt time (secs) (IQR)	54 (46–61)	50 (40.5–58.5)	40.5 (32.5–57.5)	44 (39–53)	47 (40–54)	41 (35–50)
Median total attempt time (secs) (IQR)	63 (52–74)	59 (48.5–70.5)	49 (37.5–61.5)	47.5 (43–58)	51 (43.5–58)	44 (38–52)

### Mean difference in time to successful intubation

To determine the mean difference in time to successful intubation, a subset of the data comprised of participants who were successful in intubating on attempts 1 and 2, and attempts 1 and 3, was examined. There was a statistically significant difference between groups AAB (n = 23) and ABB (n = 28) with respect to the mean difference in time taken to perform a successful intubation on attempts 1 and 2 (mean difference 11.71 seconds, 95% CI 1.95–21.47 seconds, p = 0.02). There was no significant difference between groups AAB (n = 27) and ABB (n = 27) with respect to mean difference in time taken to perform a successful intubation on attempts 1 and 3 (mean difference −2.52 seconds, 95% CI −11.64–6.61 seconds, p = 0.58). Summary values for the mean differences are shown in Table 3. Finally, there was no significant difference in success rates on the third attempt between AAB and ABB (89% vs. 86.6%, respectively, a difference of 2.4%, 95% CI 7.6–12.4%, p = 0.63).

**Table 3.** Summary data of successful intubation attempts.

Group	n	Mean difference (secs)	Standard deviation (secs)	Standard error (secs)	95% CI
**Attempts 1 and 2**					
AAB	23	15.4	16.7	3.5	8.2–22.6
ABB	28	3.7	17.9	3.4	−3.3–10.6
**Attempts 1 and 3**					
AAB	27	6.0	20.4	3.9	−2.1–14.1
ABB	27	8.5	11.5	2.2	4–13.1

### Technique

**Figure 3:**
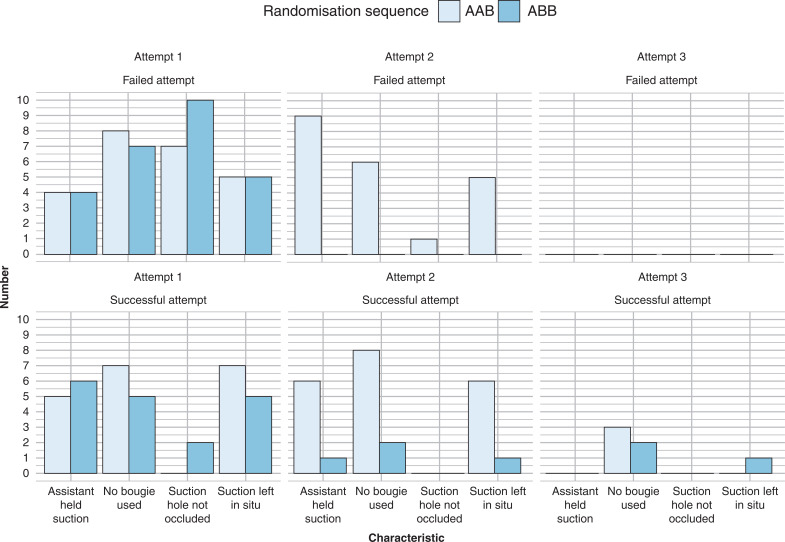
Figure 3. Techniques and omissions during intubation attempts, stratified by randomisation group, attempt number and intubation outcome.

A number of techniques were utilised by participants to facilitate intubation (Figure 3). This included asking the assistant to hold the suction catheter in the mouth (n = 35), and leaving the suction in the mouth (although without occluding the suction vent hole, n = 20). In addition, there were also instances where participants did not use a bougie (n = 48, of which 21 were successful attempts, and 27 unsuccessful) and forgot to occlude the suction vent hole on the catheter when attempting to clear the airway themselves (n = 35).

## Discussion

In this manikin study, following a brief SALAD training session, more paramedics were able to intubate a soiled airway on their first attempt, compared to those without training (90.2% vs. 53.7%, difference of 36.6%, 95% CI 24–49.1%, p < 0.001). In addition, the mean difference in time taken to perform a successful intubation between groups was statistically significant for attempts 1 and 2 (mean difference 11.71 seconds, 95% CI 1.95–21.47 seconds, p = 0.02), but not attempts 1 and 3 (mean difference −2.52 seconds, 95% CI −11.64–6.61 seconds, p = 0.58). There was no statistically significant difference in success rates on the third attempt between AAB and ABB (89.0% vs. 86.6%, difference 2.4%, 95% CI 7.6–12.4%, p = 0.63).

### Salad

While evolution of the SALAD technique has occurred as knowledge of the technique has spread, the essential principles as described by DuCanto et al. (2017) remain the same:

1. Correct positioning of the patient for intubation success (e.g. external auditory meatus level with sternal notch).
2. Holding the suction catheter (wide-bore, rigid) in a clenched-fisted right hand, with the distal end of the catheter pointing caudad and posterior, to enable manipulation of the tongue and mandible as required.
3. Leading with suction to enable identification of relevant anatomical structure (posterior portion of tongue, epiglottis, vallecular and laryngeal outlet) and following with the laryngoscope (particularly important with video laryngoscopes to avoid contaminating the optics).
4. Once the laryngoscope is in the vallecular and a view of the laryngeal inlet has been obtained, the suction catheter is ‘parked’ in the top of the oesophagus to provide continuous suction during the remainder of the intubation attempt.
5. In order to facilitate placement of the tracheal tube, the suction catheter is moved across to the left side of the mouth, ensuring that the tip remains in the oesophagus. This can be achieved either by sliding the catheter under the laryngoscope blade, or by briefly removing the catheter and inserting it to the left of the laryngoscope blade.
6. Intubating as normal, with or without a bougie.
7. Inflating the cuff on the tracheal tube to prevent further contamination of the lower airway.
8. Suctioning down the tracheal tube with a flexible suction catheter prior to ventilation.

The last step in this process typically takes 7–10 seconds to complete, a fact that was overlooked during the design of this study as it has likely confounded the mean timing differences aimed at identification learning that occurred from multiple attempts. None of the pre-training attempts finished with post-intubation suction, whereas 100% of the post-training attempts did. Successful intubations in group AAB did show timing improvements between attempts 1 and 2, but the delay in intubation completion in the latter attempts might explain why there appears to be no significant difference between attempts 1 and 3.

### Suction catheters

The suction catheters used by YAS (Pennine Healthcare Link, Yankauer 22 ch with 6 mm internal diameter tubing) have an internal diameter of approximately 6 mm and include a vent hole. For this study, the vent hole was occluded by tape for the training and post-training attempts. Failure to occlude the vent hole did occur on occasion during some attempts, and this has been reported elsewhere. Cox, Andreae, Shy, DuCanto and Strayer (2017) conducted a simulated soiled airway study with 37 emergency medicine residents, and found that 76% did not occlude the vent hole immediately on suctioning, with 60% having to be prompted to do so after 20 seconds. Catheters are available which do not contain a vent hole, which may make them more suitable for emergency situations.

Occluding the vent hole also presented a challenge for participants who left the suction in situ while continuing with an intubation attempt. While this strategy did make it easier to recommence suction when the vent hole was re-occluded by the participant, for the remainder of the attempt the suction catheter restricted the view of the oropharynx. One alternative strategy that some participants did use was to utilise the assistant to hold the suction in the oropharynx, thus maintaining continuous suction.

### Bougies

The Trust mandates the use of bougies as part of the intubation standard operating procedure. Bougies have been associated with improved first-pass intubation success (Driver et al., 2017; Kingma, Hofmeyr, Zeng, Coomarasamy, & Brainard, 2017) in other studies. In YAS, paramedics are generally taught to ‘railroad’ the tracheal tube following successful bougie insertion through the vocal cords. Stylets are not used. In this study, 48/492 (9.8%) of attempts did not utilise a bougie. This may indicate a training need, since few attempts omitted using a bougie following the SALAD training. There is a possibility that this oversight may have affected the results of the study. However, in group AAB, more participants who made a successful second intubation attempt did not use a bougie than those who were unsuccessful (8/44, 18.2% vs. 6/38, 15.8%, respectively).

### Limitations

This was a manikin study and as such, does not reflect clinical practice. For paramedics, most intubations they attempt will be at floor level and occur during a cardiac arrest, which is likely to result in some head and neck movement. The intubation attempts in the study by contrast were conducted on a static manikin at table height. In addition, the manikin could not be moved, so alternative positioning such as lateral head movement, or placing the patient in a Trendelenburg position, was not possible.

While the study did use a thickened and opaque liquid as the vomit, it did not contain any solid material, and was not as odorous as real vomit.

Finally, it was not possible to blind participants from their allocation, although they did not know that the second attempt was to be used to calculate the primary outcome. However, the researcher, acting as competent assistant, did and this may have inadvertently led to bias. In addition, for the post-training intubation attempts, it is also possible that the researcher was too proactive in assisting with suctioning down the tube at the end of the attempt, resulting in 100% of post-training attempts receiving tracheal suction.

## Conclusion

In this manikin study, following a brief training session, paramedics were able to intubate a soiled airway on their first attempt, significantly more often when using the SALAD technique.

## Acknowledgements

This study could not have been undertaken without the support and participation of paramedics working for the Yorkshire Ambulance Service NHS Trust. In addition, the study has been supported by the National Institute for Health Research Clinical Research Network: Yorkshire and Humber. Finally, thanks go to Dr Jim DuCanto for his advice and support.

## Conflict of interest

RP is editor-in-chief of the BPJ and an employee of Yorkshire Ambulance Service NHS Trust. MDT declares no conflict of interest.

## Ethics

Health Research Authority approval was obtained prior to the commencement of this study: IRAS ID: 245954. NHS Research Ethics Committee approval was not required for this study.

## Funding

This research study was funded by a College of Paramedics small research grant.

## Study registration

This study has been registered on ClinicalTrials.gov: NCT03599687. The protocol has been previously published in the *British Paramedic Journal* (Pilbery, 2018).
